# Privatisation & marketisation of post-birth care: the hidden costs for new mothers

**DOI:** 10.1186/1475-9276-11-61

**Published:** 2012-10-15

**Authors:** Cecilia Benoit, Camille Stengel, Rachel Phillips, Maria Zadoroznyj, Sarah Berry

**Affiliations:** 1 Centre for Addictions Research of BC, University of Victoria, Victoria, BC, V8W 2Y2, Canada; 2 School of Social Policy, Sociology and Social Research, University of Kent, Canterbury, CT2 7NZ, UK; 3 Centre for Addictions Research of BC, University of Victoria, Victoria, BC, V8W 2Y2, Canada; 4 Institute for Social Science Research, School of Social Science, The University of Queensland, St. Lucia, Qld, 4072, Australia; 5 Department of Sociology, McGill University, Montreal, Quebec, H3A 2T7, Canada

**Keywords:** Welfare states, Neoliberalisation, Marketisation, Familialisation, Commodification, Post-birth care, Canada

## Abstract

Retrenchment of government services has occurred across a wide range of sectors and regions. Care services, in particular, have been clawed away in the wake of fiscal policies of cost containment and neoliberal policies centred on individual responsibility and market autonomy. Such policies have included the deinstitutionalisation of care from hospitals and clinics, and early discharge from hospital, both of which are predicated on the notion that care can be provided informally within families and communities. In this paper we examine the post-birth "care crisis" that new mothers face in one region of Canada.

**Method:**

The data are drawn from a larger study of social determinants of pregnant and new mothers' health in Victoria, Canada. Mixed methods interviews were conducted among a purposive sample of women at three points in time. This paper reports data on sample characteristics, length of stay in hospital and health service gaps. This data is contextualised via a more in-depth analysis of qualitative responses from Wave 2 (4-6 weeks postpartum).

**Results:**

Out results show a significant portion of participants desired services that were not publically available to them during the post-birth period. Among those who reported a gap in care, the two most common barriers were: cost and unavailability of home care supports. Participants' open-ended responses revealed many positive features of the public health care system but also gaps in services, and economic barriers to receiving the care they wanted. The implications of these findings are discussed in relation to recent neoliberal reforms.

**Discussion & conclusions:**

While Canada may be praised for its public provision of maternity care, mothers' reports of gaps in care during the early postpartum period and increasing use of private doulas is a worrying trend. To the extent that individual mothers or families rely on the market for care provision, issues of equity and quality of care are pivotal. This paper concludes with suggestions for further research on the impact of recent changes in post-birth care on new fathers and on inequities in pre and post-birth care in less-resourced regions of the world.

## Background

Fiscal policies of cost containment in recent decades, coupled with neoliberalisation policies stressing individual responsibility and reliance on market forces, have resulted in the contraction of state provided care services in a range of countries [1]. Termed ‘de-familialising’, these establish mechanisms for the provision of care through publicly-funded social services, including housing, primary and extended health care and childcare, which are typically seen in democratic welfare states [2,3]. Reliance on market mechanisms commodifies care arrangements, transforming care into ‘products’ for purchase, and the means of care provision into specialized jobs and occupations [4]. These responses reflect the ‘care deficit’ - the situation in which the demand for care exceeds its supply [5]. The shift from publicly-funded hospital maternity care to privately-based home-based post-birth care is one example of this trend.

Research in a number of countries shows an increasing trend for maternal services to focus on infant health, and on the surveillance of parents and their parenting skills, rather than on the provision of broad ranging support to new mothers during early parenthood [6,7]. A wide range of reports over the past several years produced in the United Kingdom, for example, has centred on the notion of “early years” or “foundation years” interventions, with the aim to reduce child poverty and inequality in life chances, and to ultimately forestall persistent social problems linked to early parental neglect [8-11]. Within these reports, the importance of mothers’ mental and physical health is cited in relation to childhood health and well-being, but short-lived, rather superficial interventions such as brief visits by “health visitors”, often aimed at screening for risks, are proposed as solutions. Additionally, such visits are framed almost exclusively in terms of expected improvements in mother-child bonding (‘attachment’) and/or breastfeeding rates, and their purported consequences for early brain development and immunity, rather than any substantive improvements in the health and overall well-being of mothers themselves [11-15]. Ironically, while “parental warmth and attachment” is listed as a requirement for healthy childhood development and for forestalling social inequalities in health and overall life chances, social welfare retrenchments have reduced families’ – and specifically new mothers’ - abilities to provide such care [16].

These developments have been accompanied by major changes in the delivery of maternity services. One change is the steady increase in Cesarean-section (CS) rates. In the United Kingdom, CS rates increased from 12% in 1990–1991 reaching 23% in 2005/2006. CS rates are even higher in The United States, at 30% in 2005 [17] and Australia, where 31.5% of women gave birth by CS in 2009 [18,19]. As noted below, Canadian CS rates are also high. In contrast, Finland has seen no increase in its CS rates of 16–17% since 1994; indeed, the Finnish rate has declined slightly since 2005/2006. The World Health Organization (WHO) recommends rates of 10%-15% [20]. High CS rates suggest elective intervention, and while they are an important birth option, they are also linked to increased risks for maternal and infant morbidity, and therefore should not be practiced in excess of evidence based guidelines [21].

Another change in the delivery of maternity services is decreasing lengths of hospital stay post-birth. In the past two decades there has been a shift as cultural norms about the need for a ‘lying in’ period have eroded [22]. From a standard hospital lying-in period of between 1-2 weeks for uncomplicated vaginal birth in the 1950s, to recent length of post-birth in Canada, United Kingdom, United States (US), Sweden and Australia is now around two to three days or less [23]. Indeed, in the US by the early 1990s, the lying-in period had taken on a “drive- through” character as hospital stays of 12 to 24 hours for uncomplicated vaginal births, and 48 to 72 hours for uncomplicated CS births have become standard [24]. As with rising CS rates, there has been considerable controversy surrounding the question of whether earlier discharge of mothers and babies is safe and linked to positive health outcomes [25].

To date, the implications of these trends in the direction of the privatisation and commodification of post-birth care have not been evaluated from the point of view of the main recipients of care. In this paper, we explore the hidden costs of these developments for new mothers in one Canadian city. Below we provide a background of recent changes in post-birth care in Canada and an overview of our methods. This is followed by a presentation of descriptive characteristics of our research participants, length of stay in hospital and health service gaps. We contexualise our data via a more in-depth analysis of qualitative responses from new mothers. We then discuss our results in light of recent trends in the privatization and marketisation of post-birth care, and make suggestions for further research on the impact of recent changes in post-birth care on new fathers, and on inequities in pre- and post-birth care in less-resourced regions of the world.

### Post-birth care in Canada

Costs of maternity services in Canada, including salaries for service providers, are paid for through general taxes and included as public services under the country’s universal health care program, Medicare. Virtually all pregnant women give birth in hospitals and receive maternity care from obstetricians or family physicians, although professional midwives and nurse practitioners have emerged as autonomous providers since the mid-1990s.

Similar to many other countries, Canada has revamped its reproductive care services in recent decades [26]. The length of time Canadian women spend in hospital following childbirth has decreased dramatically, from five-seven days in the 1960s to between 24 to 48 hours after vaginal delivery in the current decade [27]. As noted earlier, this trend has taken place at the same time that the CS rates have steadily increased, with total national CS increasing from 17.6% in 1995, to 21.1% in 2000 and 25.6% in 2004. The variation in CS rates are almost double across the provinces/territories. In British Columbia (BC), while Saskatchewan (20.8%) and Manitoba (19.8%) had among the lowest rate. BC has the second highest rate in Canada at 30% [28]. In BC (2005), Vancouver Island Health Authority (where our study took place – see below) had the highest CS rate of 32% and the Northern Health Authority had the lowest rate of 26% [29]. Furthermore, while the CS rate for mothers age 40 or older is currently double (42%) the rate for mothers age 20 to 24 (21%), there is little evidence that this variation is based on mothers’ demand -- the so called “too posh to push” argument [30]. Changes in obstetric practice and neoliberal reorganization of health care environments are among the main reasons for increased CS rates among Canadian mothers across all age groups [31].

In addition to women being discharged earlier to private home environments, Canada’s public health care system currently covers a narrow range of post-birth care services. Typically, where post-birth care services exist, they are comprised of surveillance and referral, rather than more substantive and intensive home-based services [32]. Post-birth care services vary substantially by region, and federal involvement is limited to the provision of informational supports for the provinces and the publication of national guidelines for maternity and newborn care [33]. Publicly-funded post-birth care following discharge from a hospital involve a single home visit from a public health nurse; indeed, in some regions this service has been reduced to a single telephone call.

On the positive side, publicly funded midwifery services have become available for care throughout pregnancy, birth and post-birth. After considerable public debate and advocacy by consumer organizations, in the mid-1990s midwifery became institutionalized and publicly-funded initially in the province of Ontario, with BC following soon thereafter. Today, the midwifery option is available in seven regions in roughly half of the provinces in Canada [34]. Midwives hold a university bachelor’s degree through one of the newly-established direct-entry (non-nursing prerequisite) programs and are certified by the provincial/territorial Colleges of Midwives to work as a primary care provider during pregnancy, labour and delivery, and the immediate post-partum period. Midwifery care is thus a viable option for pregnant women in many regions and their midwifery services, including post-birth care services, are reimbursed through the public purse.

Yet the impact of this midwifery expansion to date has been small. In fact, less than 5% of births in Canada are currently attended by a certified midwife [35]. While the percentage is higher in some provinces, including British Columbia (11% midwifery attended deliveries in 2011) where our study was conducted [36], a substantial proportion of women in all parts of the country who want to see a midwife are currently unable to find one. Many pregnant women and their families must pay for their care out of pocket for both pre- and post-birth care from privately-practicing midwives. Further complicating access to midwifery services is the fact that, even where such services are publicly funded, less-educated women, younger mothers, women without a partner, indigenous women, and women living in rural and remote areas or socioeconomically disadvantaged communities, are less likely to have access to midwifery care during pregnancy, labour and delivery and in the post-birth period [37].

As publicly-funded and delivered post-birth care services have contracted, a wide range of services for purchase on the market has grown to fill the burgeoning care gap. While these services have garnered media attention in Canada [38], there currently exist no published research studies on the for-profit postnatal services that have emerged to fill in the post-birth care gap. Post-birth doulas advertise and deliver a vast array of high-intensity, practical services and supports, including newborn care, breast- and bottle-feeding support, child-minding services, meal preparation, household chores and management (including laundry, plant, and pet care services), errand-running, and peer support/counselling. These providers advertise a similarly wide range of degrees in areas such as nursing and midwifery, and/or have completed specialized training as post-birth doulas and lactation consultants. Unlike the interventions offered though state-directed programs, commodified forms of post-birth care appear to be carefully and flexibly tailored to the unique needs of each woman, and can be contracted out for extended periods of time (i.e., an overnight stay, or an entire week). As Carroll and Reiger (2005: 101) state: these postmodern consultants fill “a specific role not only within maternity care institutions, but in private practice in the community” [39].

But such privately-delivered post-natal care tends to be quite expensive, making such support accessible only to those who are able to pay for it. Providers who advertise online generally charge around $25(CAD) on a per-hour basis, or anywhere from $100 to $1000(CAD) for overnight or week-long package deals, respectively. No information currently exists on user demographics, patterns of use, or outcomes associated with these forms of commodified care, though such information would offer insight into the types and levels of unmet needs that exist.

In sum, the provision of post-birth care in Canada is stratified by geographical location, social status factors, and capacity to pay for services on the market [26,39,40]. Rising CS rates, early discharge from hospital and limited state-provided post-care support, especially in jurisdictions without public provision of midwifery, leave new mothers and their families with two main alternatives: to rely on their own resources for care provision, or to rely on the market for the purchase of care services. Next we explore these challenges for new mothers in one Canadian city.

## Methods

### Study design and data collection strategies

The data analyzed in this paper are drawn from a larger study of social determinants of pregnant and new mothers’ health in one urban region of BC, Canada. A key objective of the project was to comparatively examine the experiences of mothers under physicians’ and midwives’ care. Our sample selection was theoretically-informed and based on two overarching criteria: (i) diversity of backgrounds, and (ii) choice of maternity care provider. Our purposive sample included pregnant women who represented a range of ages, ethnicities, educational levels, parity and economic status and had chosen either a certified midwife or physician for their primary attendant. The proportion of women choosing to have the care of a midwife is thus artificially high compared to the general population, which estimates put at 25% in the local region, a utilization rate higher than that of any other city in Canada [41]. We attained our sample by distributing posters and flyers to places that pregnant women frequent in the Victoria Census Metropolitan Area (CMA), including physicians’ and midwives’ offices, pre-natal classes, single-parent resource centres and low-income outreach programs. While our non-random sampling technique precludes us from knowing if our findings can be generalized to the broader regional population, we believe that our sample reflects the diversity of social and economic backgrounds and the style of maternity care available through the public health care system in the area (see Table 1). 

**Table 1:** Selected characteristics, study population compared to the Victoria Census Metropolitan Area (CMA), 2006. Income is in Canadian dollars (CAD)

**Measure**	**Sample population**	**Victoria CMA**
Aboriginal background	4.5%	2.8% (Female)
Visible minorities	11.2%	9.0% (Females)
Average (mean) annual income Gross household income	$52565 $53,500 (median)	$66,594 $59,015 (median)
Own home	40.4%	61.8% (by household, not specific for age or gender)

One-hundred and six women responded to our research postings, with an estimated population-based recruitment rate of 3.5%. We completed interviews with 93 women any time during their third trimester of pregnancy (wave 1), 89 at 4 – 6 weeks post-birth (wave 2), and 83 during the 4 – 6 months post-birth period (wave 3). Thirteen participants were lost at each stage because they became unavailable for an interview or had scheduling conflicts, had a therapeutic abortion or still birth, or moved outside the region and were not accessible by telephone. Our final participant retention rate for wave 3 was 89 percent (n=83). Our analysis of the 13 participants who were not included in the study due to attrition or missing/incomplete data shows that they had a lower mean income and education level than the others.

Just under half (n=42) of wave 1 participants were under the care of a certified midwife and just over half received care from either a maternity physician or obstetrician (n=51). The four interviewers involved in data collection collaborated during pre-testing of the instruments and interviewer training to ensure consistency of delivery. The majority of interviews were conducted in participants’ homes; others occurred at places of convenience to participants, such as research offices and coffee shops. Whenever possible, the same interviewer conducted all three of a participant’s interviews to facilitate rapport. We were interested in hearing the women’s own voices about their pregnancies, birth experiences and post-birth care options and gaps, and so the interviews included both closed and open-ended questions. In addition to collecting standard demographic information, questions focused on a variety of health related topics such as health service utilization, indicators of physical and mental health, and experiences of pregnancy and early motherhood including sources of stress and social support. To facilitate triangulation of data on topics of central interest within the study (birth, parenting, care, and health experiences), the interviews included both closed and open-ended questions.

A feature of our mixed-methods design was to follow many of our closed-ended questions with a probe asking participants to explain their response, and then asking them an open-ended follow-up question. The two questions relevant to our analysis are: *How satisfied were you with the post-birth care you received from paid care providers from the time of your baby's birth, up to the present? Would you have wanted any of these types of care, but they were not accessible to you? If so, what were the barriers to your accessing this care?* We believe this design that mixes quantitative and qualitative data collection methods is richer than exploring this subject with only one method. This is because it enables us to quantify and qualify key variables, giving us an opportunity to triangulate both types of data on topics of interest and to elaborate on the meaning and experiences subsumed within survey statistics. Comparing data from the different methods also helps to determine the validity of measures, providing insight for design innovations in follow-up studies. In addition, after having completed key survey questions, participants were keen to elaborate on what factors they had considered when choosing their survey answer and to narrate stories that exemplified their experience. Thus, we believe this instrument design is more respondent-receptive in practice. We delivered potentially sensitive closed- and open-ended questions, including those on income and depression, using a self-administered, written questionnaire completed by the participant at the end of the face-to-face interview. All questions were read aloud by the interviewer, with the exception of self-administered items and the interviews were tape-recorded. At the completion of the self-administered portion of the interview, participants placed their answers in an envelope, sealed the contents and returned the data to the interviewer. This process allowed for extra assurance of confidentiality and anonymity of the participants, as the self-administered portion was reviewed at a later date. Responses to closed-ended questions were entered and analyzed using SPSS 12.0 software.

In the case of this paper, the qualitative data were analyzed using the following procedures. The second author initially coded the relevant transcribed transcriptions, and the third author repeated this exercise and independently identified the central themes in the answers for each question. Based on an analysis of the lists they arrived at independently, the two authors drew a third list of common themes by question. The first author then reviewed the list of themes and a final version was made. The transcriptions were subsequently coded thematically by the second author and a few transcriptions were coded by two of the first and third authors. These transcriptions were compared for coding consistency/reliability. Our study was approved by the Human Research Ethics Board at the University of Victoria, Canada.

## Results

### Sample characteristics of participants

Our descriptive characteristics are based on the subsample of wave 2 participants (n=89) (interviewed 4-6 weeks post birth) who gave complete answers on questions regarding their access to support and care in the post-birth care period. As shown in Table 1, participants were slightly more likely to identify being of Aboriginal or visible minority background, and to have lower income and lower home ownership than the population in the study region [42]. Participants had somewhat higher levels of high school completion compared to the local population. The lower income and home ownership of the sample is likely a reflection of their younger, childbearing age.

Participants’ median number of days in hospital was 2.4 days overall, and 1.9 days for persons reporting a vaginal birth; these findings support the literature noted earlier regarding a reduction in length of hospital stays following the birth of a child compared to earlier generations. While the demedicalisation of birth, as evidenced by short term hospital stays, may not in and of itself signal a lack of care in the post-birth period, coupled with the finding that approximately one third of participants (33.8%) reported that they desired post-birth care services that were not available, it is not clear that reductions in hospital stays have been accompanied by increases in community-based care options. The closed-ended data also show that greater proportion of participants with incomes below the median for the census region reported they would have liked post-birth care that was unavailable to them (39.5%) compared to participants with an income above the median (27.3%). The most commonly cited barriers to obtaining post birth support services included “cost” (42.8%) and “unavailability of home care supports” (38%).

As noted above, we asked participants to expand on the topic of access to post-birth care services, and barriers to receiving the care they wanted. The next section of the paper focuses on themes that emerged, both in terms of participants’ satisfaction with access to post-birth care, as well as lack of access to the services they wanted to help them through the early stages of being a new mother.

### Qualitative analysis

#### Satisfaction with post-birth public services

Several participants either did not identify any additional post-birth care needs, or specifically described aspects of Canadian post-care services that contributed to a positive experience during the first six weeks of the birth of their baby. Most of these participants noted practitioners and services that contributed to quality continuity of care. Sarah,^a^ age 27 and a mom for the third time, described her satisfaction with her maternity doctor’s team: “Just wonderful people, they’re just, they’re awesome. They really listen; they never treat me like an overactive, over-reactive mother. You know they always take my opinion very seriously and uh, they’re just really caring and really great.” Another participant, Annie, expressed gratitude for the public health nurse who visited her post-birth:

"The nurse contacted me right away when I got out of the hospital and she came to check up on me. [I]t’s kinda nice to know that they’ll come to you. [Because], you know, when you first get out of the hospital and especially after a C-section [you are] sore. You don’t really wanna go anywhere; you just wanna be home. So it’s nice to have that for them to come to you. (age 34; 1^st^ child)"

Annie’s response addresses the importance of accessibility to health care during the post-birth period and having services available that are flexible to the needs and physical capacity of the new mother. Many participants mentioned positive aspects of post-birth care also highlighted easily-accessible helpful information. This included the 24-hour nurse hotline, which they saw as a beneficial support system for answering questions. Participants praised the workers of the hotline service as “knowledgeable” and “helpful” and easily accessible by phone.

Other participants discussed the benefit of having a midwife for their most recent child. Kathy explained:

"I went with the midwife this time round [and] I just felt that I was, I felt really, really well looked after […] it’s just so different having, you know, two women midwives where that’s all they do, versus the GP who does all kinds of things and doesn’t specialize in […] the amount of time, I think that’s a huge thing that they, the midwives, offer. (age 35; 2nd child)"

Another participant, Myra, highlighted that in addition to the more lengthy visits offered by midwives in comparison to physicians, an additional benefit was the availability of in home postpartum care and flexible appointment times:

"I can call them [the midwives] anytime and they will come over. Like it’s not even, it’s never a question like – if I ever needed to get in to see them now, like it was – they came here in the first two weeks and then I’ve been going there and if I needed to get in, I know they would just squeeze me in and - so that’s why I like it. (age 25; 2nd child)"

The dedicated time that the midwives’ spent on these participants as well as the flexibility with regard to service time and location were greatly valued in the post-birth period. The support and focus of midwifery care resulted in satisfactory continuity of care and specific support for the woman based on the midwives’ expertise. The qualities that made up a positive labour, delivery and post-birth experience were the same qualities addressed as absent from other participants’ post-birth experiences.

#### Dissatisfaction with post-birth services covered under the health care system

A major theme articulated by participants’ who expressed dissatisfaction with their interaction with the health care system was poor care during the labour and delivery period, and a lack of follow-up during the post-birth period. Concerning her hospital experience, Jessica stated:

"You really are like a number in the hospital. They’re, you don’t, they’re not a lot of caring women and I’m not sure whether it’s because they’re tired of their job, you know and they’re not happy that way or whether they’re just you know, but there were a couple of kind women there, you know […] you just, you don’t really get that attention that I really believe that you deserve and you need. (age 36; 3rd child)"

A second time mother, Lena, age 31, stated “I kinda *(sic\* fell through the cracks a bit with this second baby… I don’t know what but nobody called me afterwards to remind me of things like shots and so I actually went, I went far too long before I got his shots.” Several respondents commented on a feeling that health care providers “did not pay attention, had a “million other things to do”, and that it would be beneficial if they could spend more time with the patient”.

Some participants identified a need for more emotional and social support services, in particular supports that were not connected to risk assessment activities. The desire for this type of support came from a variety of avenues, including health care professionals, mental health services and a space to informally socialize with other mothers. Kelsey, age 22 and a first time mom, expressed a desire to have a health care professional to confide in while her son was in the hospital that would not “potentially deem me ‘unfit’ to have my baby because I’m depressed or something […] that just scared me so I, I guess I had access to somebody to talk to but I didn’t use it because I felt it probably caused more trouble than I would have wanted.”

Women in Canada, including in the study area, are routinely screened for postpartum depression as part of public health care services [43]. Using the Beck Depression Inventory, we found that 15% of participants at Wave 2 and 21% of participants at Wave 3 reported moderate depression symptoms [44]. Some new mothers, such as Kelsey, who identify the need for mental health services during the post-birth period do not access the desired services because they are concerned that doing so will undermine the perception of competency and capacity to care for a child.

#### What women wanted and was not publicly available

As noted the above, thirty-four % of women wanted additional services than were provided via the health care system but were not available due to varying factors. Many of these women lacked a strong informal support system and the income to purchase post-care services out-of-pocket. Theresa, a first-time mother of twins, remarked:

"Well since I’ve been home like there have been times when having the two has just been really, really intense and really hard. I’m getting a grip on it now, but there were times within that first six weeks that I just felt like I was gonna lose my mind. It would be really nice if there was somehow just a number you could call, in the community, just to, I don’t know, like listen to you for a minute or, or, I don’t know, rush over and hold one of your babies! (age 30; 1^st^ child)"

Sabina wished that the public health nurse would provide more continuous care during the initial weeks after the birth. But this is not a covered public care service in the study region, and as a result she had to go without:

"I would have liked the public nurse to come back again because she said she was going to and she didn’t because I had breastfeeding questions and you know he had that acne, that um, from breastfeeding and I didn’t, I didn’t, wasn’t sure whether that what it was or not. Just questions that you have. (age 30; 1^st^ child) "

Three-quarters (74.7%) of the new mothers in our study reported that someone, primarily friends and family members, came forward to help them during the post-birth period; mothers appearing to be the most common source of informal. Without such care, they would have had to purchase it on the market, as Becky, age 33 and entering motherhood a second time, stated: “If, if I didn’t have my, my sister and mum, I definitely probably would consider a doula.” Other participants who wanted more post-birth care sought out a private doula, a service which is not publicly funded. Tina was able to pay for her doula, but saw it as an expense that she should not have to pay for:

"It would have been nice if the, you know, the doula. Like, we paid quite a bit for the doula. [Y]ou know it’s expensive uh, anyway it would be nice if there was um public health care. I think they’re worth it, it just like, it was something that we paid for and we knew wanted the support so but definitely it was expensive. (Age 24; 1^st^ child)"

Other participants who did not have access to family care or the needed economic resources to pay for a doula went without. As Barb, age 23 and a 1^st^ time mom, noted: “I would have liked a doula but they’re very expensive.”

Breastfeeding advice has become more commonplace in Canadian hospitals and all midwives are trained to routinely give such advice. At wave 2, 64.8% of new mothers were breastfeeding exclusively, but that drops to 50% at Wave 3. Eighty-nine per cent of the women reported problems with breastfeeding their infants at 3-6 weeks postpartum; At wave 3, 35% of them were still reporting breastfeeding problems. We asked the women who responded “yes” to having breastfeeding difficulties what sorts of difficulties they had. Some women had multiple problems at once, while others had one or two, and the level of severity of each source of difficulty also varied among participants. The most prominent of these were: sore nipples (the top source of difficulty), difficulty latching on, sleepy baby, and milk undersupply. Karli expressed her concerns about breastfeeding, stating:

"When we left the hospital I kinda worried because I still wasn’t, you know, breastfeeding that… My milk hadn’t come in and there was things like that so um. It was fine in the hospital cuz I had that support but once you go home you don’t have that support any longer. (age 31; 1^st^ child)"

Other respondents similarly commented that “breastfeeding was the hardest” and that it would have been beneficial to have a lactation consultant who had time and expertise to devote specifically to the task. As these participants note, lactation consultation is a very important service as new mothers have a short window of time to establish breastfeeding before they may turn to bottle/formula feeding because they are worried that the baby is not being adequately nourished. The public health nurse visit may not be sufficiently timely or intensive enough to meet this important need. In fact, a lactation consultant is even a good addition to midwifery care in the first two weeks as the midwives help with breastfeeding but some people, particularly first time mothers, require more intensive support in early post-birth period. In our study only one-quarter (25.8%) of participants mentioned they had used the services of a lactation consultant in the post-birth care period.

## Discussion

In this paper we examined the provision of post-birth care for mothers in one Canadian city. The findings presented here indicate that the majority of participants were happy with their post-care services, noting that they contributed to a positive experience during the first six weeks of the birth of their baby. Most of these participants noted practitioners and services that contributed to quality continuity of care. However, a minority of the women in our study expressed a desire for post-birth care services that were not available to them locally. Many of these women reported incomes below the median for the census region. Unavailability of home care supports were at the top of their list of barriers to access this post-birth care services they desired.

Post-birth care intersects the line between public and private spheres, and blurs the state’s responsibility towards women in need of such services [45]. The adoption of neoliberal reform ideologies in countries such as Canada but also in Australia has further pushed government responsibility away from post-natal women and towards their families and informal support networks [26]. This push results in a greater burden for women without the financial or support networks to secure appropriate post-birth care. New mothers who do not have secure informal social supports or the means to pay for services out of pocket – one-third of our sample - often fall through the safety net in these contexts.

The cultural politics of post-birth care are premised on the often mistaken assumption that women need little if any care after their discharge from hospital, an understanding which is, in turn, based on very narrow conceptions of care itself [45]. Where government-based home care is provided within these contexts, it is generally limited in scope, fragmented in terms of its provision across multiple carers, and differs in type and quality between hospitals and geographic regions [45-47]. To the extent that community-based services exist, they tend to focus on the infant rather than the mother, or on a form of surveillance aimed at identifying ‘at risk’ groups. In some developed welfare states, such as Sweden and the Netherlands, the two branches of the welfare state have cooperated and integrated to such an extent that they are able to meet the care needs of women in the post-birth period [26,48-50]. In other countries, including the US, Australia, the UK and Canada, these branches do not closely overlap. It is within the latter contexts that a care gap exists. In some of these countries, midwives and family networks provide adequate social care to some new mothers but these tend to be women who are more advantaged. The market – through the private services of doulas, lactation consultants, etc. is variously called upon to fill the care gap [51]. To the extent that individual mothers or families rely on the market for care provision, issues of equity and quality of care are pivotal [50,52].

The importance of post-birth care extends beyond the immediate need of providing women with the support after the birth of their children. Setting women up with adequate and appropriate supports in such a way that minimizes the financial and emotional stress can not only assist with better health outcomes for the mother, but also for her infant during the crucial period after birth. While policy documents in other high income countries like England stress the importance of reducing inequity during the foundational years of a child’s life, the health and well-being of the mother in the post-birth period is lacking in this discussion [8-11]. As seen in the participants’ lived experiences of their pregnancy and post-birth care in BC, mothers’ and children’s welfare in the post-natal period is often closely connected. Appropriate provincial-assisted care from government services that attend to the needs of post-natal women in turn can aid with the creation of a stable, reduced-stress environment for children during an important phase of their life. Currently, the potential of the postnatal period as a period of health promotion opportunity is not being fully realized and additional supports for new parents, including those described by respondents, which consider both parent and infant health promotion should be considered.

### Concluding remarks and future research

This study is not without important limitations. Our single case study of post-birth care in one Canadian city does not allow us to generalize to the situation elsewhere. The non-random nature of our sample and its limited size also prevented us from ascertaining whether the current care deficit has an especially negative impact on new mothers who are disadvantaged due to inadequate housing, single parenthood, younger age, Indigenous background, racial minority status, or chronic illness. Further research with a larger and more diverse sample is needed to fill in these gaps. It was not within the scope of this paper to investigate the impact of recent changes in post-birth care on new fathers [53]. This will be a crucially important next step in laying the groundwork for future research on the gendered underpinnings of postnatal care provision [54]. Finally, this paper has focused attention on inequities in one dimension of reproductive care services in the economically-resourced regions of the world [55]. Research in lower-income countries point to the need for immediate action on far more entrenched inequities in pre and post-birth care [56].

## Endnotes

^a^ Pseudonyms are used to protect the identity of the participants.

## Competing interests

The authors declare no competing interests concerning this paper.

## Authors’ contributions

The first author planned the study, and oversaw the data collection. The first three authors performed the analysis. All authors contributed to the drafting of the initial manuscript, and read and approved the final version.
